# 4-Cyano­anilinium perchlorate

**DOI:** 10.1107/S1600536808030687

**Published:** 2008-09-27

**Authors:** Jing Dai

**Affiliations:** a Ordered Matter Science Research Center, College of Chemistry and Chemical Engineering, Southeast University, Nanjing 210096, People’s Republic of China

## Abstract

The title compound, C_7_H_7_N_2_^+^·ClO_4_^−^, comprises discrete ions which are inter­connected by N—H⋯O hydrogen bonds, leading to a neutral one-dimensional network along the [100] direction.

## Related literature

For the chemistry of nitrile derivatives, see: Xiong *et al.* (2002 ▶); Jin *et al.* (1994 ▶); Brewis *et al.* (2003 ▶); Fu *et al.* (2008 ▶); Duncia *et al.* (1991 ▶); Fu & Zhao (2007 ▶); Dai & Fu (2008 ▶); Smith *et al.* (2000 ▶).
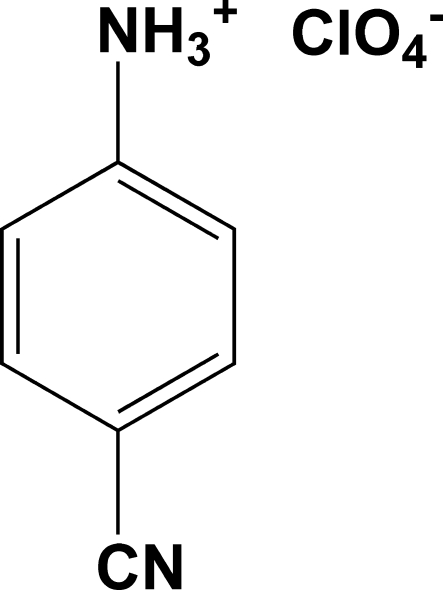


## Experimental

### 

#### Crystal data

C_7_H_7_N_2_^+^·ClO_4_^−^
*M* *_r_* = 218.60
Triclinic, 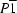

*a* = 4.9905 (10) Å
*b* = 6.9465 (14) Å
*c* = 13.998 (3) Å
α = 94.87 (3)°
β = 95.68 (3)°
γ = 103.99 (3)°
*V* = 465.57 (17) Å^3^
*Z* = 2
Mo *K*α radiation
μ = 0.40 mm^−1^
*T* = 298 (2) K
0.25 × 0.15 × 0.15 mm

#### Data collection

Rigaku Mercury2 diffractometer
Absorption correction: multi-scan (*CrystalClear*; Rigaku, 2005 ▶) *T*_min_ = 0.941, *T*_max_ = 1.000 (expected range = 0.886–0.942)
4861 measured reflections
2126 independent reflections
1851 reflections with *I* > 2σ(*I*)
*R*_int_ = 0.023

#### Refinement

*R*[*F* ^2^ > 2σ(*F* ^2^)] = 0.036
*wR*(*F* ^2^) = 0.097
*S* = 1.05
2126 reflections
128 parameters
H-atom parameters constrained
Δρ_max_ = 0.25 e Å^−3^
Δρ_min_ = −0.34 e Å^−3^

### 

Data collection: *CrystalClear* (Rigaku, 2005 ▶); cell refinement: *CrystalClear*; data reduction: *CrystalClear*; program(s) used to solve structure: *SHELXS97* (Sheldrick, 2008 ▶); program(s) used to refine structure: *SHELXL97* (Sheldrick, 2008 ▶); molecular graphics: *SHELXTL* (Sheldrick, 2008 ▶) and *PLATON* (Spek, 2003 ▶); software used to prepare material for publication: *SHELXTL*.

## Supplementary Material

Crystal structure: contains datablocks I, global. DOI: 10.1107/S1600536808030687/bx2181sup1.cif

Structure factors: contains datablocks I. DOI: 10.1107/S1600536808030687/bx2181Isup2.hkl

Additional supplementary materials:  crystallographic information; 3D view; checkCIF report

## Figures and Tables

**Table 1:** Hydrogen-bond geometry (Å, °)

*D*—H⋯*A*	*D*—H	H⋯*A*	*D*⋯*A*	*D*—H⋯*A*
N1—H1*A*⋯O2^i^	0.89	2.04	2.881 (2)	158
N1—H1*B*⋯O3^ii^	0.89	1.98	2.855 (2)	166
N1—H1*C*⋯O4^iii^	0.89	2.04	2.871 (2)	156

Symmetry codes: (i) 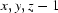; (ii) 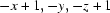; (iii) 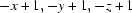.

**Fig. 1.**
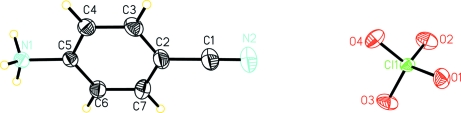
A view of the title compound with the atomic numbering scheme. Displacement ellipsoids were drawn at the 30% probability level.

**Fig. 2.**
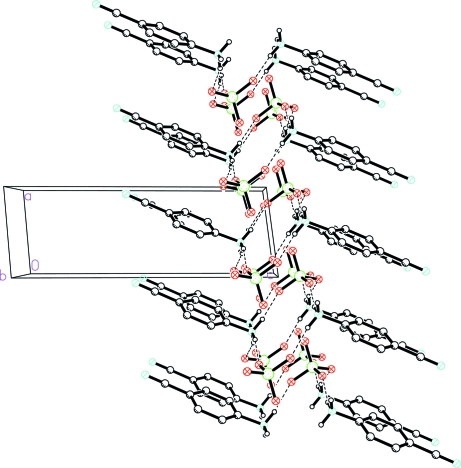
The crystal packing of the title compound viewed along the b axis and all hydrogen atoms not involved in hydrogen bonding (dashed lines) were omitted for clarity.

## supplementary crystallographic information

### Comment

Nitrile derivatives have found wide range of applications in industry and coordination chemistry as ligands. For example, phthalonitriles have been used as starting materials for phthalocyanines (Jin *et al.*, 1994), which are important components for dyes, pigments, gas sensors, optical limiters and liquid crystals, and which are also used in medicine, as singlet oxygen photosensitisers for photodynamic therapy (Brewis *et al.*, 2003). And nitrile compounds are the precursor of tetrazole complexes (Duncia *et al.*, 1991; Xiong *et al.*, 2002; Fu *et al.*, 2008). Recently, a series of benzonitrile compounds have been reported (Fu & Zhao, 2007; Dai & Fu, 2008; Smith *et al.*, 2000). As an extension of these work on the structural characterization, we report here the crystal structure of the title compound *p*-cyanoanilinium perchloride. The crystal data show that in the title compound, the N1 atom of the amine group is protonated. The nitrile group and the benzene ring are essentially coplanar. The C1≡N2 bond length of 1.135 (3) Å is within the normal range (Fig. 1).The crystal packing is stablized by cation–anion N—H···O hydrogen bonds, building an infinite one-dimensional chain parallel to the *a* axis. (Table 1, Fig. 2).

### Experimental

*p*-cyanoaniline (3 mmol, 354 mg) was dissolved in the solution of distilled water (10 ml) and perchloride acid (0.5 ml), and evaporated in the air affording colorless block crystals of this compound suitable for X-ray analysis were obtained.

### Refinement

All H atoms attached to C and N atoms were fixed geometrically and treated as riding with C—H = 0.93 Å (aromatic) and N—H = 0.89 Å with *U*_iso_(H) = 1.2Ueq(C or N).

### Crystal data

**Table:**

C_7_H_7_N_2_^+^·ClO_4_^−^	*Z* = 2
*M**_r_* = 218.60	*F*(000) = 224
Triclinic, *P*1	*D*_x_ = 1.559 Mg m^−^^3^
Hall symbol: -P 1	Mo *K*α radiation, λ = 0.71073 Å
*a* = 4.9905 (10) Å	Cell parameters from 1806 reflections
*b* = 6.9465 (14) Å	θ = 3.0–27.5°
*c* = 13.998 (3) Å	µ = 0.40 mm^−^^1^
α = 94.87 (3)°	*T* = 298 K
β = 95.68 (3)°	Block, colourless
γ = 103.99 (3)°	0.25 × 0.15 × 0.15 mm
*V* = 465.57 (17) Å^3^	

### Data collection

**Table:**

Rigaku Mercury2 diffractometer	2126 independent reflections
Radiation source: fine-focus sealed tube	1851 reflections with *I* > 2σ(*I*)
graphite	*R*_int_ = 0.023
Detector resolution: 13.6612 pixels mm^-1^	θ_max_ = 27.5°, θ_min_ = 3.0°
ω scans	*h* = −6→6
Absorption correction: multi-scan (CrystalClear; Rigaku, 2005)	*k* = −8→8
*T*_min_ = 0.941, *T*_max_ = 1.000	*l* = −18→18
4861 measured reflections	

### Refinement

**Table:**

Refinement on *F*^2^	Primary atom site location: structure-invariant direct methods
Least-squares matrix: full	Secondary atom site location: difference Fourier map
*R*[*F*^2^ > 2σ(*F*^2^)] = 0.036	Hydrogen site location: inferred from neighbouring sites
*wR*(*F*^2^) = 0.097	H-atom parameters constrained
*S* = 1.05	*w* = 1/[σ^2^(*F*_o_^2^) + (0.0483*P*)^2^ + 0.1253*P*] where *P* = (*F*_o_^2^ + 2*F*_c_^2^)/3
2126 reflections	(Δ/σ)_max_ < 0.001
128 parameters	Δρ_max_ = 0.25 e Å^−^^3^
0 restraints	Δρ_min_ = −0.34 e Å^−^^3^

### Special details

**Table:**

Geometry. All e.s.d.'s (except the e.s.d. in the dihedral angle between two l.s. planes) are estimated using the full covariance matrix. The cell e.s.d.'s are taken into account individually in the estimation of e.s.d.'s in distances, angles and torsion angles; correlations between e.s.d.'s in cell parameters are only used when they are defined by crystal symmetry. An approximate (isotropic) treatment of cell e.s.d.'s is used for estimating e.s.d.'s involving l.s. planes.
Refinement. Refinement of *F*^2^ against ALL reflections. The weighted *R*-factor *wR* and goodness of fit *S* are based on *F*^2^, conventional *R*-factors *R* are based on *F*, with *F* set to zero for negative *F*^2^. The threshold expression of *F*^2^ > σ(*F*^2^) is used only for calculating *R*-factors(gt) *etc*. and is not relevant to the choice of reflections for refinement. *R*-factors based on *F*^2^ are statistically about twice as large as those based on *F*, and *R*- factors based on ALL data will be even larger.

### Fractional atomic coordinates and isotropic or equivalent isotropic displacement parameters (Å^2^)

**Table:**

	*x*	*y*	*z*	*U*_iso_*/*U*_eq_	
N1	0.6157 (3)	0.2508 (2)	0.12597 (11)	0.0399 (4)	
H1A	0.4771	0.2405	0.0791	0.048*	
H1B	0.6876	0.1460	0.1180	0.048*	
H1C	0.7468	0.3623	0.1238	0.048*	
N2	0.0727 (7)	0.2647 (4)	0.5503 (2)	0.1058 (10)	
C1	0.1672 (6)	0.2642 (4)	0.47983 (19)	0.0743 (8)	
C2	0.2877 (5)	0.2620 (3)	0.38998 (16)	0.0549 (5)	
C3	0.2722 (5)	0.4100 (3)	0.33016 (17)	0.0557 (5)	
H3	0.1860	0.5101	0.3478	0.067*	
C4	0.3859 (4)	0.4064 (3)	0.24466 (15)	0.0458 (4)	
H4	0.3789	0.5049	0.2041	0.055*	
C5	0.5097 (3)	0.2566 (3)	0.21961 (13)	0.0362 (4)	
C6	0.5293 (4)	0.1094 (3)	0.27829 (14)	0.0464 (5)	
H6	0.6169	0.0104	0.2604	0.056*	
C7	0.4158 (5)	0.1127 (4)	0.36408 (16)	0.0574 (6)	
H7	0.4252	0.0145	0.4046	0.069*	
Cl1	−0.00044 (8)	0.22266 (6)	0.91125 (3)	0.03315 (14)	
O1	−0.2849 (3)	0.1812 (2)	0.92711 (11)	0.0552 (4)	
O2	0.1712 (3)	0.3237 (2)	0.99794 (12)	0.0598 (4)	
O3	0.0665 (3)	0.0398 (2)	0.88369 (13)	0.0593 (4)	
O4	0.0420 (4)	0.3478 (2)	0.83610 (12)	0.0668 (5)	

### Atomic displacement parameters (Å^2^)

**Table:**

	*U*^11^	*U*^22^	*U*^33^	*U*^12^	*U*^13^	*U*^23^
N1	0.0442 (8)	0.0392 (8)	0.0403 (9)	0.0162 (7)	0.0100 (6)	0.0048 (6)
N2	0.141 (3)	0.0996 (19)	0.0746 (17)	0.0122 (17)	0.0625 (17)	−0.0038 (14)
C1	0.0839 (18)	0.0741 (17)	0.0597 (16)	0.0059 (14)	0.0296 (13)	−0.0047 (13)
C2	0.0545 (12)	0.0599 (13)	0.0450 (12)	0.0039 (10)	0.0167 (9)	−0.0053 (10)
C3	0.0560 (13)	0.0514 (12)	0.0617 (14)	0.0159 (10)	0.0210 (10)	−0.0056 (10)
C4	0.0485 (11)	0.0424 (10)	0.0506 (12)	0.0168 (8)	0.0126 (9)	0.0046 (8)
C5	0.0328 (8)	0.0375 (9)	0.0366 (9)	0.0068 (7)	0.0046 (7)	0.0004 (7)
C6	0.0547 (11)	0.0455 (10)	0.0441 (11)	0.0205 (9)	0.0097 (9)	0.0072 (8)
C7	0.0714 (15)	0.0609 (13)	0.0425 (12)	0.0163 (11)	0.0126 (10)	0.0140 (10)
Cl1	0.0306 (2)	0.0284 (2)	0.0426 (3)	0.00948 (15)	0.00733 (15)	0.00697 (15)
O1	0.0333 (7)	0.0667 (9)	0.0627 (10)	0.0079 (6)	0.0146 (6)	−0.0046 (7)
O2	0.0531 (9)	0.0534 (9)	0.0661 (10)	0.0147 (7)	−0.0150 (7)	−0.0090 (7)
O3	0.0595 (9)	0.0396 (7)	0.0839 (12)	0.0271 (7)	0.0058 (8)	−0.0036 (7)
O4	0.0880 (12)	0.0539 (9)	0.0623 (11)	0.0127 (8)	0.0217 (9)	0.0293 (8)

### Geometric parameters (Å, °)

**Table:**

N1—C5	1.462 (2)	C4—C5	1.371 (3)
N1—H1A	0.8900	C4—H4	0.9300
N1—H1B	0.8900	C5—C6	1.380 (3)
N1—H1C	0.8900	C6—C7	1.378 (3)
N2—C1	1.135 (3)	C6—H6	0.9300
C1—C2	1.447 (3)	C7—H7	0.9300
C2—C7	1.385 (3)	Cl1—O4	1.4202 (15)
C2—C3	1.392 (3)	Cl1—O3	1.4215 (14)
C3—C4	1.375 (3)	Cl1—O1	1.4222 (14)
C3—H3	0.9300	Cl1—O2	1.4346 (16)
			
C5—N1—H1A	109.5	C4—C5—C6	122.22 (18)
C5—N1—H1B	109.5	C4—C5—N1	118.25 (16)
H1A—N1—H1B	109.5	C6—C5—N1	119.50 (16)
C5—N1—H1C	109.5	C7—C6—C5	118.39 (19)
H1A—N1—H1C	109.5	C7—C6—H6	120.8
H1B—N1—H1C	109.5	C5—C6—H6	120.8
N2—C1—C2	179.6 (3)	C6—C7—C2	120.1 (2)
C7—C2—C3	120.66 (19)	C6—C7—H7	120.0
C7—C2—C1	119.9 (2)	C2—C7—H7	120.0
C3—C2—C1	119.4 (2)	O4—Cl1—O3	109.51 (11)
C4—C3—C2	119.1 (2)	O4—Cl1—O1	109.25 (11)
C4—C3—H3	120.4	O3—Cl1—O1	108.89 (10)
C2—C3—H3	120.4	O4—Cl1—O2	109.01 (11)
C5—C4—C3	119.54 (19)	O3—Cl1—O2	110.69 (10)
C5—C4—H4	120.2	O1—Cl1—O2	109.47 (10)
C3—C4—H4	120.2		
			
C7—C2—C3—C4	0.0 (3)	C4—C5—C6—C7	1.1 (3)
C1—C2—C3—C4	−179.7 (2)	N1—C5—C6—C7	−176.89 (19)
C2—C3—C4—C5	0.6 (3)	C5—C6—C7—C2	−0.4 (3)
C3—C4—C5—C6	−1.2 (3)	C3—C2—C7—C6	−0.1 (4)
C3—C4—C5—N1	176.83 (18)	C1—C2—C7—C6	179.6 (2)

### Hydrogen-bond geometry (Å, °)

**Table:**

*D*—H···*A*	*D*—H	H···*A*	*D*···*A*	*D*—H···*A*
N1—H1A···O2^i^	0.89	2.04	2.881 (2)	158.
N1—H1B···O3^ii^	0.89	1.98	2.855 (2)	166.
N1—H1C···O4^iii^	0.89	2.04	2.871 (2)	156.

Symmetry codes: (i) *x*, *y*, *z*−1; (ii) −*x*+1, −*y*, −*z*+1; (iii) −*x*+1, −*y*+1, −*z*+1.
